# An IoT-Based Life Cycle Assessment Platform of Wind Turbines

**DOI:** 10.3390/s21041233

**Published:** 2021-02-09

**Authors:** Jinjing An, Zhuo Zou, Guoping Chen, Yaojie Sun, Ran Liu, Lirong Zheng

**Affiliations:** 1 School of Information Science and Technology, Fudan University, Shanghai 200433, China; jjan15@fudan.edu.cn (J.A.); zhuo@fudan.edu.cn (Z.Z.); yjsun@fudan.edu.cn (Y.S.); rliu@fudan.edu.cn (R.L.); 2 Project Management Department, Wuxi Institute of Fudan University, Wuxi 214131, China

**Keywords:** wind turbine, Internet of Things (IoT), life cycle assessment (LCA)

## Abstract

Life cycle assessment (LCA) is conducive to the change in the wind power industry management model and is beneficial to the green design of products. Nowadays, none of the LCA systems are for wind turbines and the concept of Internet of Things (IoT) in LCA is quite a new idea. In this paper, a four-layer LCA platform of wind turbines based on IoT architecture is designed and discussed. In the data transmission layer, intelligent sensing of wind turbines can be achieved and their status and location can be monitored. In the data transmission layer, the LCA platform can be effectively integrated with enterprise information systems through the object name service (ONS) and directory service (DS). In the platform layer, a model based on IMPACT 2002+ is developed, and four management modules are designed. In the application layer, different from other systems, energy payback time (EPBT) is selected as an important evaluation index for wind turbines. Compared with the existing LCA systems, the proposed system is specifically for wind turbines and can collect data in real-time, leading to improved accuracy and response time.

## 1. Introduction

Wind energy is a type of important clean and renewable energy with abundant reserves. As a green and environmental protection power generation technology, wind turbines can effectively reduce environmental pollution. In recent years, due to the gradual maturity of wind power technology and the continuous decline in the cost of wind power generation, wind turbines have been widely used in the world. According to “BP Statistical Review of World Energy June 2019”, the annual growth rate of global wind farms in 2018 is 12.6%, and the power generation capacity is 1170.0 TWH. Additionally, the rated power of wind turbines has developed from dozens of kilowatts to megawatts [1]. Large-scale wind turbines are considered to be the most affordable in all renewable energy sources [2]. Nowadays, most of the research focuses on the financial aspects of wind farms or the optimization of the wind power generation process [3]. In the upstream of wind power generation, high energy consumption and large environmental emissions are produced due to the usage of fossil fuels and building materials and the manufacturing of other power generation equipment. Therefore, it is important to analyze the environmental benefits of wind turbines by comparing their energy produced with energy consumption and pollution to the environment, to ensure that they provide a net environmental benefit.

As a technology to evaluate the environmental factors related to products and their potential impacts, life cycle assessment (LCA) has been developed as an important tool to evaluate the energy and environmental performance of products in their life cycle. The LCA of wind turbines can not only help enterprises carry out green design or improvement of the key energy consumption parts but also help the wind power industry change from the terminal-oriented management mode to the full life cycle management mode. In order to minimize energy consumption and environmental emissions, it is necessary to optimize energy management and carry out green design through accurate LCA of wind turbines. Moreover, the resource and environmental costs and energy conservation and emission reduction potential can be obtained through quantifying energy consumption and pollutant emissions. On this basis, suggestions for green and low-carbon management strategies of the wind power industry can be put forward.

The accuracy of LCA mainly depends on the accuracy of the life cycle inventory and the rationality of the environmental analysis process. In the current research, the inventory data come from investigation, literature, or the existing database, which are universal to a certain extent and can be used in different products. However, due to the effectiveness and regionality of inventory data, the input and output data of the same material will vary greatly in different regions with different process levels. Consequently, the difficulty mainly lies in the acquisition and accuracy of data, because of the polymorphism, time variability, and sensitivity of LCA data.

The existing problems in the life cycle assessment of wind turbines are as follows:

1. The existing life cycle assessment process mainly relies on software system databases, which are from the average data of various industries, or manually collecting from literature or on site. The real time, dynamic, and accuracy of evaluation data are far from satisfactory. Therefore, the results of LCA are not accurate enough.
2. The existing LCA software are commercial, and they are for many products. As a result, the process is not specifically for wind turbines.
3. The existing LCA methods mainly include problem-oriented midpoint methods and damage-oriented endpoint methods, having their limitations.
4. The existing LCA systems and enterprise information systems (EIS) are independent of each other and cannot be effectively integrated together, and the data of enterprise information systems cannot be effectively utilized.

As a new and rapidly developing technology, the Internet of Things (IoT) holds great promise in handling the abovementioned issues. IoT is a pervasive presence around us of a variety of things or objects (such as radio-frequency identification (RFID) tags, sensors, actuators, mobile phones, etc.), which can interact with each other and cooperate with their neighbors through unique addressing schemes [4,5]. IoT is recognized as one of the most promising networking paradigms due to bridging the gap between the cyber and physical worlds [6]. Its characteristics include comprehensive perception, reliable transmission, and intelligent data processing. Because of providing interoperability, compatibility, and reliability in the world, standardization is also one of the important factors for the widespread application of the Internet of things [7]. Although IoT has been successfully applied in many fields, the application in LCA is still in its infancy. Therefore, it is necessary to study systematically and thoroughly the potential applications of IoT in LCA of wind turbines, which is of great significance.

In order to solve the above problems, based on IoT, this paper proposed a new LCA platform for wind turbines. Additionally, the main innovations are as follows:

1. The IoT technology is applied to achieve the real-time, intelligent collection of energy consumption and environmental impact data in the entire life cycle of wind turbines. Therefore, the databases of the platform have the function of updating and expanding, which ensures the objectivity and accuracy of the assessment results. Additionally, the status and location of wind turbines and their components can be monitored with the support of IoT technology.
2. A novel LCA architecture based on IoT is proposed, which achieves the energy consumption assessment and environmental impact assessment of wind turbine from parts to products, including the stages of design, raw material acquisition, production and manufacturing, transportation and installation, operation and maintenance, and recovery and disposal.
3. An LCA model for wind turbines is proposed based on IMPACT 2002+ [8], which combined the midpoint method with the endpoint method. Compared with other methods, the proposed model reduces the complexity and uncertainty of the evaluation process.
4. Based on ONS and DS of IoT technology, the effective integration method between the proposed platform and the existing EIS is innovated, thereby constructing an open and extensible platform for life cycle assessment.

The remainder of the paper is organized as follows. Section 2 introduces the background of LCA and presents related work about LCA of wind turbines. In Section 3, the architecture of the proposed platform based on IoT is described. A prototype system is developed in Section 4. Additionally, the characteristics of the system are discussed in Section 5. The conclusion and future work are presented in Section 6.

## 2. Background and Related Work

### 2.1. Life Cycle Assessment (LCA)

#### 2.1.1. Development of LCA

**Figure 1:**
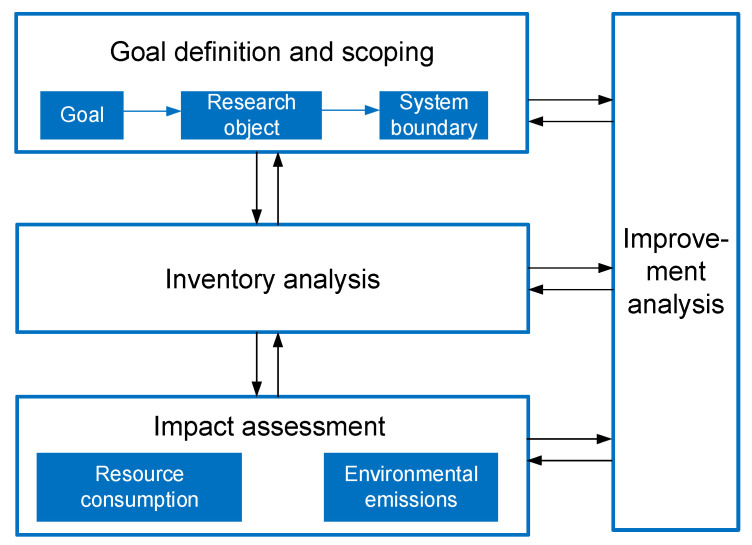
The framework of life cycle assessment (LCA) with four steps.

Life cycle assessment (LCA) has made remarkable progress, since the first life-cycle-oriented methods were proposed in the 1960s. Currently, LCA is defined as a tool that assesses the potential environmental impacts and resource consumption in a product’s life cycle, i.e., from raw material acquisition, via production and use stages, to waste management [9]. The general methodological framework and standards of LCA are defined by ISO 14040 and ISO 14044 [10,11]. It includes the following four steps: goal definition and scoping, inventory analysis, impact assessment, and improvement analysis, as shown in Figure 1.

Currently, the research on LCA mainly focuses on the evaluation of the new energy system, product resource consumption, and environmental emission. Additionally, LCA has been widely used in various fields, including materials, energy sources, transportation and construction, etc. Boyden et al. compared the environmental impact of three management strategies for lithium-ion batteries in Australia ehydro-metallurgy, pyro-metallurgy, and landfill through LCA [12]. Geng et al. used content analysis to investigate various patterns in existing LCA studies in the building industry [13]. Wulf et al. studied the assessment of the life cycle of hydrogen production from biomass for transportation purposes concerning greenhouse gas emissions, emissions with an acidification potential and the fossil energy demand [14]. Foelster et al. explored the environmental impacts of a refrigerator recycling system in Brazil and quantified its ecological advantages over primary resource production through LCA [15].

The methods of LCA mainly include problem-oriented midpoint methods and damage-oriented endpoint methods [16]. Additionally, midpoint methods focus on the intuitive interpretation of the impact of products on the environment, while endpoint methods mainly focus on the damage caused by the consequences to human health, environment, and resources. With the application of LCA in various industries, its methodology system is also developed. At present, the midpoint methods include CML-IA, TRACI 1.0, EDIP 2003, TRACI 2, and so on; the endpoint methods include EPS 2000, Eco-Indicator99, LIME 2.0 (2008), LC-Impact, and so on, and the methods combining both include IMPACT 2002+, ReCiPe 2008, ILCD/PEF/OEF, and IMPACT World+ [17]. The above methods have been widely used, but each has its own limitations. 

In recent years, in view of the continuous expansion and complexity of evaluation objects, LCA methods are also developed in new forms. Additionally, the combination of LCA methodology with other methods has become a common practice. Suhariyanto et al. proposed a multi-life cycle assessment perspective for assessing the environmental impacts on the multiple life cycle product system [18]. Bai et al. proposed an integrated LCA-based decision-support platform named HIT.WATER scheme, linking the currently available LCA system with a water quality model, Plackett–Burman design, and conjoint analysis [19]. Yao et al. analyzed waste mobile phone management and recycling by using an integrated method of LCA and system dynamic prediction [20]. Morbidoni et al. developed the CAD-integrated LCA tools to support the simplified LCA method, which could be used as eco-design tools [21].

#### 2.1.2. Software of LCA

LCA needs a large amount of data as the basis and a variety of models as tools. In order to improve the research efficiency, the software developed includes Simapro, Gabi, LCAiT, eBalance, PEMS, and so on.

- Simapro is developed by the University of Leiden, the Netherlands. It provides abundant databases and a variety of evaluation methods. The database in the manufacturing phase is the most detailed. It is also the most popular LCA software in the world.
- GaBi is developed by Institut fur Kunststoffprufung und Kunst—stoffkunde from Stuttgart, Germany. It has the latest wide-range comprehensive database and provides sensitivity analysis.
- LCAiT (LCA Inventory Tool) is developed by Chalmers Industrieknik in Göteborg, Sweden. Although it has fewer own databases, it can connect with external databases. It is suitable for people who understand the flow of material and energy.
- eBalance is developed by IKE Environmental Technology Co. Ltd. from Chengdu, China. It includes three databases: Chinese Life Cycle Database, European Reference Life Cycle Database, and Ecoinvent Database. It supports China’s localized evaluation of energy conservation and emission reduction.
- Pira Environmental Management System (PEMS) is developed by Pira International, UK. The parameters in the database are from Europe, which cannot be modified or edited.

Based on the system boundary, the main input of the above software includes the consumption of various resources (metal, minerals, water, etc.) and different types of energy (such as electricity, coal, natural gas, gasoline, diesel, etc.). Based on the establishment of process flow and the database in the software, the energy consumption and environmental emissions (such as wastewater, waste solid, CO_2_, CO, SO_2_, Nox, COD, etc.) in each stage can be calculated.

#### 2.1.3. Quantification of LCA

According to the framework of ISO14040 standard, the process of LCA quantification includes calculation of environmental impact potential, standardization, and weight assessment. 

The environmental impact potential refers to the sum of environmental emissions or resource consumption in the whole life cycle of a product, which can be expressed as follows:

(1) $$EP(j)=\sum EP{\left(j\right)}_{i}=\sum [Q{\left(j\right)}_{i}\cdot EF{\left(j\right)}_{i}]$$

where EP(j) is the environmental impact potential j, j = {global warming, acidification, eutrophication, ozone depletion, solid waste, hazardous waste, etc.}, $EP{\left(j\right)}_{i}$ is the contribution of substance i to the environmental impact potential j, $Q{\left(j\right)}_{i}$ is the emissions of substance i, and $EF{\left(j\right)}_{i}$ is the equivalence factor of the substance i to the environmental impact potential j. The equivalence factor is usually calculated based on one kind of substance, such as CO_2_ for global warming and SO_2_ for acidification.

In order to compare and analyze different types of environmental impact, it is necessary to carry out standardization. The expression is as follows:

(2) $$NEP(j)=\frac{EP(j)}{ER(j)}$$

where NEP(j) is the potential environmental impact and resource consumption after standardization, and ER(j) is the benchmark of environmental impact potential j, which varies with time and region.

In order to analyze and evaluate different environmental impact types more reasonably, it is necessary to carry out weight evaluation, which can be expressed as follows:

(3) WP(j)=WF(j)×NEP(j)

where WP(j) is the potential environmental impact after weighting, and WF(j) is the weight coefficient of environmental impact potential j.

For a product composed of multiple components, the environmental impact potential of the product is the sum of the environmental impact potential of all the components, which is calculated by

(4) $$EP(j)=\sum_{x}EP_{x}(j)=\sum_{x}\left[EP_{{}_{x}}^{D}(j)+EP_{{}_{x}}^{A}(j)+EP_{{}_{x}}^{M}(j)+EP_{{}_{x}}^{T}(j)+EP_{{}_{x}}^{I}(j)+EP_{{}_{x}}^{O}(j)+EP_{{}_{x}}^{R}(j\right)]$$

where EP(j) refers to the environmental impact potential j of the product, $EP_{x}(j)$ indicates the environmental impact potential j of the component x, and $EP_{{}_{x}}^{D}(j)$, $EP_{{}_{x}}^{A}(j)$, $EP_{{}_{x}}^{M}(j)$, $EP_{{}_{x}}^{T}(j)$, $EP_{{}_{x}}^{I}(j)$, $EP_{{}_{x}}^{O}(j)$, and $EP_{{}_{x}}^{R}(j)$ indicate the environmental impact potential j of the component x in the stage of design, raw material acquisition, production and manufacturing, transportation, installation, operation and maintenance, and recovery and disposal, respectively.

### 2.2. LCA of Wind Turbines

In recent years, a number of papers related to the life cycle assessment of wind energy have been published. Some of them are as follows.

Demir et al. [22] used the LCA method to compare and analyze wind turbines with 50, 80 and 100 m hub heights in Turkey. Additionally, the results show that the high-power wind turbine with high hub heights has a low environmental load and high energy return rate. Uddin et al. [23] studied wind turbines from three aspects of energy utilization, emission reduction, and environmental impact through LCA. Yang et al. [24] constructed a hybrid LCA model to facilitate the accounting of the energy consumption and greenhouse gas emission of the first offshore wind farm in China. Lloberas-Valls et al. [25] illustrated a detailed “cradle-to-gate” life cycle assessment of the 15 MW wind turbines by using GaBi 6 commercial software and Ecoinvent 2.2 databases. Gomaa et al. [26] investigate the environmental impact and energy performance of wind farms in the southern region of Jordan using an LCA method. Jesuina et al. [27] attempted to quantify the relative contribution of individual stages toward life cycle impacts by conducting a life cycle assessment with SimaPro and the IMPACT2002+ method. Moghadam et al. [28] developed an in-depth LCA evaluation of three different drive train choices based on permanent-magnet synchronous generator technology for 10 MW offshore wind turbines. Mendecka et al. [29] proposed simplified LCA models that predict the final results with acceptable uncertainty. Additionally, the obtained simplified LCA models were generalized for different site-specific wind conditions. Martinez et al. [30] created an LCA model of the repowering process of an old wind farm with low power wind turbines and promoted an analysis from the point of view of the potential environmental impact and benefit of a wind farm repowering process. The organizational life cycle assessment of a service provider for photovoltaic and wind energy projects was carried out in the United Kingdom [31]. The environmental impacts of hydropower generation, nuclear, and wind were analyzed, assessed, and compared in China through a comprehensive life cycle assessment approach [32]. Crawford [33] presented the results of a life cycle energy and greenhouse emissions analysis of two wind turbines and considers the effect of wind turbine size on energy yield.

### 2.3. IoT in LCA 

With the rapid development of IoT technology, it has become a promising technology that can make product management more flexible and efficient [34]. According to the survey, it is noticed that the application of IoT has not caught the researchers’ attention enough in the field of LCA. Additionally, only a few studies about life cycle assessment based on IoT have been developed. IoT has been employed for collecting real-time data at each stage of the product to achieve the multi-structure as well as multi-stage carbon emission evaluation [35]. By applying LCA and IoT, Du et al. achieved efficient and intelligent management of renewable resources and discussed the environmental effects of various types of renewable resources [36]. Tao et al. [37] investigated the specific application of the Internet of things in each stage of product energy management. An LCA method for energy-saving and emission-reduction based on IoT and bill of material was designed and presented [38].

## 3. System Design

Before designing the LCA system of wind turbines, it is necessary to carry out goal definition and scoping, because it is directly related to the complexity of LCA evaluation of wind turbine and the accuracy of evaluation results.

### 3.1. Goal Definition and Scoping

#### 3.1.1. Goal Definition

For different users, the goals of LCA in this platform are divided into the following three types. (1) For enterprises, the improvement schemes are proposed for the stages or parts with large energy consumption/environmental impact, and improvement measures of green design of wind turbine will be provided. (2) For the government, the reference standard of the research object will be established through the LCA results of each stage, and the energy consumption and environmental emission standards of each stage will be established. (3) For the third-party testing and certification department, it is to carry out green certification for the wind turbine by comparing the evaluation results with the defined green index value of the wind turbine.

#### 3.1.2. Research Objects

The related research objects are mainly divided into three types: wind farm, wind turbine, and its main components. Wind farm refers to the place where wind energy is captured, converted into electric energy, and sent to the power grid through transmission lines, including wind turbines, power collection lines, substations, and roads. A wind turbine is mainly composed of a rotor, an engine room, a tower, and a foundation. For the main components, the rotor is mainly composed of blade, hub, nose cone, and bolt; the engine room is usually integrated by engine room frame, which covers generator, gearbox, transformer, electrical, and mechanical equipment; tower supports engine room and rotor, mainly made of steel, aluminum, copper, and plastic; foundation provides ground support for all the above parts, which is composed of concrete, steel, and iron.

Usually, for enterprises, the research object is mainly wind turbine or its main parts; for the third-party testing and certification department, the research object is wind farm or wind turbine or its main components; for the government, the research object is usually wind farm or wind turbine. The complexity of LCA decreases in the order of wind farm, wind turbine, and its main components.

#### 3.1.3. System Boundary

The system boundary is determined according to the research objectives. The wider the research scope is, the more the relative workload will be. Nowadays, the system boundary of LCA pays more attention to evaluation efficiency instead of breadth and depth, which is excluding the stage not related to the research objectives from the system boundary after comprehensively considering the accuracy of the evaluation results and the complexity of the evaluation work, and determining the most efficient system boundary according to the needs of research objectives. For example, when enterprises intend to improve the green design of wind turbines, they only need to study the stages of raw material acquisition, manufacturing, recycling, and scrapping, while the transportation, on-site installation, operation, and maintenance stages have little impact on the research objectives can be ignored.

For different users, the objectives, and scope of LCA of wind turbines are shown in Table 1.

**Figure 2:**
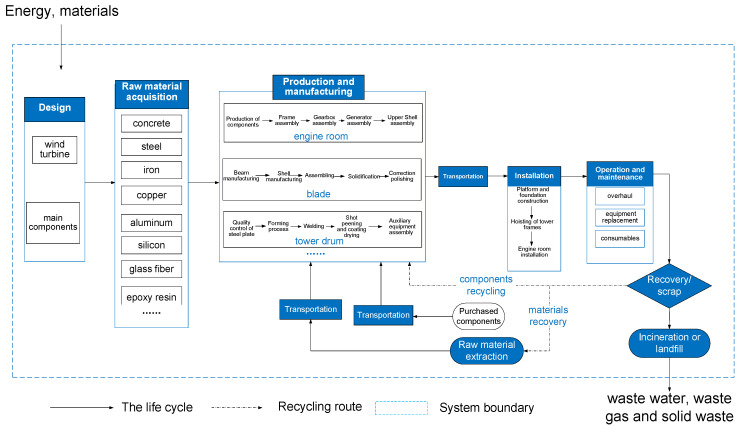
The boundary of the LCA system for the wind turbine.

The evaluation of the wind turbine is the most common in practical applications. Therefore, this system is mainly designed for wind turbines. Taking wind turbine as an example, the system boundary of LCA for wind turbines is shown in Figure 2, which includes the design stage, raw material acquisition stage, production and manufacturing stage, transportation and installation stage, operation and maintenance stage, and recovery and disposal stage.

### 3.2. Architecture of the LCA Platform 

**Figure 3:**
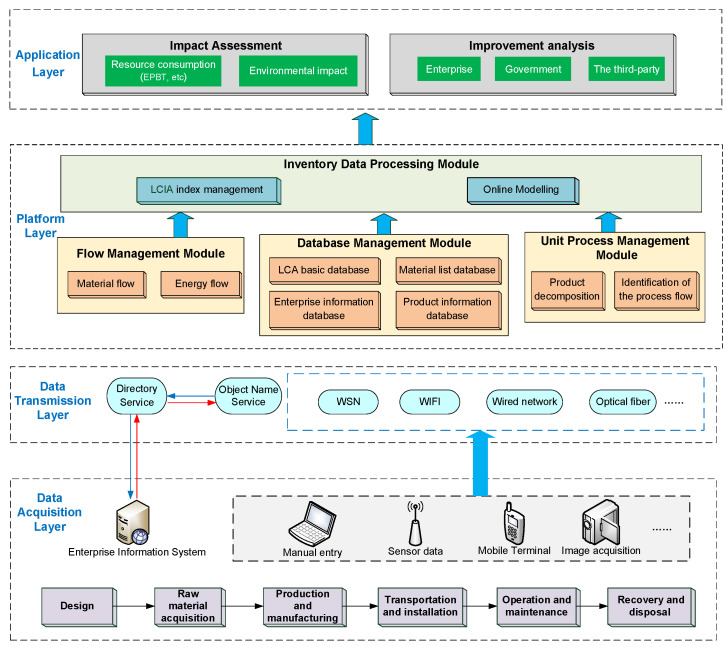
The architecture of IoT-based LCA platform of wind turbines.

Through the analysis of the objectives and scope of LCA of wind turbines, the architecture of the LCA platform based on IoT technology is constructed. The data acquisition layer, data transmission layer, platform layer, and application layer are included. The architecture of the LCA platform for wind turbines is shown in Figure 3.

Each layer of the platform is introduced as follows.

### 3.3. Data Acquisition Layer

In the data acquisition layer, intelligent identification, positioning, tracking, monitoring, and management can be achieved with the help of RFID devices, barcode identification equipment, global positioning systems, laser scanners, and other information sensing equipment. Consequently, all the input and output data involved in the life cycle of wind turbines will be obtained and preprocessed. 

There are three ways for collecting data: (1) By using RFID, wireless, mobile, and sensor equipment, real-time and intelligent collection of energy consumption and environmental emission data can be generated from different material units and manufacturing processes of wind turbines. (2) Bill of Materials can be acquired from the information management systems of enterprises. (3) For the data that cannot be collected through sensors or obtained by EIS, they can be collected by manual input, such as enterprise fundamental information, some parameters and specifications of wind turbines, etc. In the process of manual entry, it is necessary to ensure the authenticity and effectiveness of data and make sure there is evidence to follow for data output in the later stage.

The data acquiring methods in each stage of wind turbines are as follows. 

#### 3.3.1. Design Stage

In the design stage, the wind turbine and its main components are designed, and the three-dimensional models of them are established, and the structure is tested and analyzed. Because the design tools such as computers and other electronic equipment are mainly used in this stage, the energy consumption and environmental emission data are not generated directly. Therefore, the energy consumption is measured by smart meters, and other data can be obtained through EIS. All the design schemes have a great impact on the final results.

#### 3.3.2. Raw Material Acquisition Stage

Raw materials include parts, components, semi-manufactures, etc. Raw materials can be obtained in two ways: production in the factory and purchased from other factories. For the raw materials produced in the factory, which usually including internal and external blades and baffles of wind turbines, relief valve, brake, transmission shaft, rod, bearing, clutch, flying pendulum, cover, and so on, the relevant data can be obtained by integrating with EIS. For the purchased raw materials, which usually including tire coupling, permanent magnet synchronous generator, standard parts, etc., the relevant data are collected by reading barcodes or RFID tags attached to them.

The elementary raw materials in the wind turbine include concrete, steel, iron, copper, glass fiber, epoxy resin, etc., as shown in Table 2. The relevant data can be collected from the related database.

#### 3.3.3. Production and Manufacturing Stage

In the production and manufacturing stage, the energy consumption data of water, electricity, and gas are collected by embedded systems and sensing equipment installed on manufacturing equipment, and environmental emission data can be obtained from intelligent sensors and environmental monitoring systems. At the same time, in order to improve the reliability of the data, the measurement calibration certificates of relevant monitoring equipment should be upload simultaneously to the enterprise information database in the platform layer. According to the material composition of each component and the energy consumption and environmental emissions of various main raw materials, the total energy consumption and environmental emissions of wind turbines can be calculated in this stage.

#### 3.3.4. Transportation and Installation Stage

In the transportation stage, the fuel consumption, weight of product transported, and transportation distance are collected by sensors on vehicles, and the total energy consumption and pollutant emission of the transportation process can be calculated based on the fuel consumption per kilometer. In the installation stage, which includes the installation of foundation construction, tower hoisting, and engine room, the data related to mechanical equipment can be obtained by sensors, and other data can be manually entered.

#### 3.3.5. Operation and Maintenance Stage

In the operation stage, the environmental information (such as air temperature, air pressure, wind speed, etc.) and its operation data (such as rotor speed, power, etc.) can be collected through sensors. In addition, equipment calibration records (such as power transmitter, current transformer, anemometer, temperature, and humidity meter, etc.) need to be uploaded in time. Based on IoT technology, the wind turbine status can be continuously monitored, and detection automation can be achieved. In the maintenance stage, energy consumption is mainly generated by using various maintenance tools or component replacement. Additionally, its data can be collected by reading RFID tags or manually entering.

#### 3.3.6. Recovery and Disposal Stage

This stage is the process of wind turbine disassembly, renovation, recycling, reuse, reassembly, or disposal. Recovery methods include direct recovery and recovery after solvent extraction. Scrapping methods of the wind turbine include open stacking, landfill, and incineration. Eighty-five to ninety percent of the foundation, tower, and engine room components of the wind turbine can be recycled. However, it is difficult to recover the blades, because the thermosetting materials cannot be degraded and the cost of recovering fiber materials is very high [39]. Nowadays, there are three methods for blades recycling: (1) the blades are disassembled, and the materials are reused for municipal construction and other fields; (2) the blades are broken and added into the construction materials after recycling; (3) recycle the chemical materials and reuse them after decomposition of the blades. The data in this stage are mainly collected by environmental monitoring sensors, and it can also be obtained from LCA basic databases, such as an energy warehouse and a material database.

### 3.4. Data Transmission Layer

The data transmission layer can also be called the network layer, which transfers the data captured in the data acquisition to the servers in the platform layer through networking technologies including WSN, WIFI, Ethernet, and so on. In addition, this paper proposes to achieve effective integration with EIS through object name service (ONS) and directory service (DS). As the infrastructure of the network layer, the ONS and DS provide external services.

The ONS is defined by GS1 EPCglobal, and it is the core technology of the Internet [40]. In 2013, the GS1 published the ONS2.0 standard that enables the use of GS1 Identification Keys. Furthermore, ONS 2.0 introduces the Federated Model to solve the cooperating name service problem [41]. 

The ONS mainly completes the network resource analysis pointing request of all kinds of IoT identification and can map the Code identification to the resource entrance. The ONS can be called by the applications that need to conduct the name service. The DS is mainly deployed to offer the index access to EIS, which is at the data acquisition layer. Each enterprise submits event indexes automatically to their representative DS server. Before adopting this integration scheme, enterprises need to register and maintain their domain names in the enterprise information database in the Database Management Module in advance.

**Figure 4:**
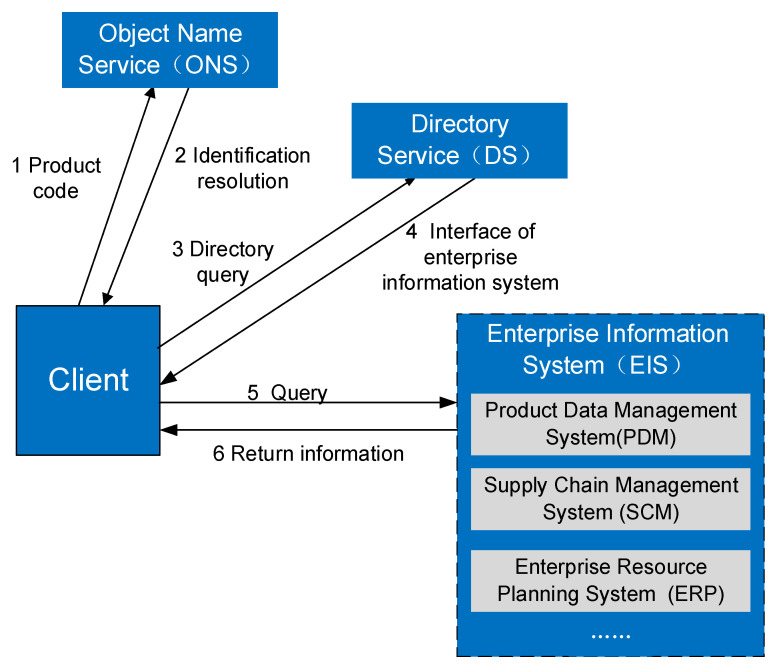
Integration solution with enterprise information systems (EIS).

The process of integration with enterprise information systems is shown in Figure 4.

As shown in Figure 4, first of all, the user queries the product code. Secondly, the system analyzes the product identification and feeds back the results to users by ONS, which is the resource entry. Then, the user queries the directory and the service interface of the EIS can be accessed through the DS. The user queries the required information through the interface next. Finally, effective integration with EIS can be achieved, including PDM, ERP, SCM, and so on.

### 3.5. Platform Layer

The platform layer mainly carries out an inventory analysis of the life cycle of wind turbines. Inventory analysis is the process of collecting and objectifying the basic data of all relevant processes of wind turbines, which mainly includes the analysis of raw material consumption, production and manufacturing energy consumption, material and product transportation energy consumption, operation and maintenance energy consumption, and scrapping treatment environmental emissions. The integrity and accuracy of the data obtained by inventory analysis affect the accuracy of LCA results directly. The platform layer includes four modules: Unit Process Management Module, Flow Management Module, Database Management Module, and Inventory Data Processing Module.

#### 3.5.1. Unit Process Management Module

Unit process refers to the most basic unit in life cycle assessment. In this module, the decomposition of the research object and the identification of the process flow are completed. In the meantime, in order to quantitatively determine the input and output corresponding data, users need to input the unit process attribute data of the research object in this module, and manage all the unit processes on this basis.

The structure of wind turbines is complex, and there are many kinds of components and materials. Decomposing the wind turbine and classifying the materials and its components play a crucial role in the process of inventory analysis, which will affect the accuracy of the evaluation results. In our system, the analytic hierarchy process (AHP) [42] is used to determine the optimal decomposition path of the research object.

**Figure 5:**
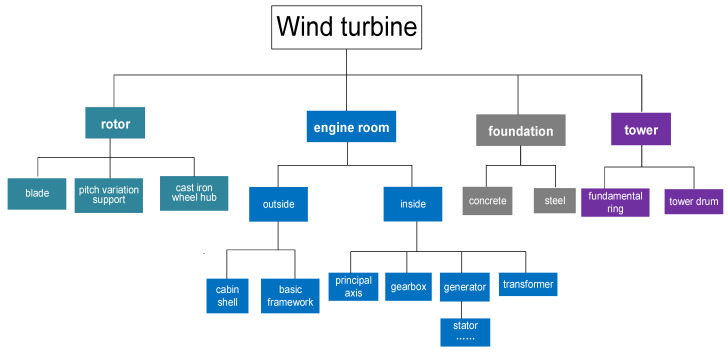
Decomposition of a wind turbine.

An example of wind turbine decomposition is shown in Figure 5.

LCA focuses on the full life cycle and goes deep into each process to analyze the input and output data. For this reason, it is also necessary to identify the key influencing factors of each stage of the process flow. On this basis, the data of each basic unit are input and output, and the data of each stage are classified and summarized. The main LCA influencing factors of wind turbines in each stage are shown in Table 3.

#### 3.5.2. Flow Management Module

Material flow management and energy flow management are included in this module. Material flow and energy flow data are a vital link and are used to record the material/energy interaction between different unit processes. In this module, the material flow and energy flow information of each unit can be added, modified, deleted, and viewed by users, which provides support for inventory data processing of the wind turbine. 

#### 3.5.3. Database Management Module

The Database Management Module is an important module to support the completion of the life cycle assessment of wind turbines. It mainly includes the functions of data addition, data storage, and data calls. For users, the required data can be obtained from the database conveniently, timely, and accurately.

Four databases are included in this module, which are as follows:

- LCA basic database: the existing mature database, including Ecoinvent, European Reference Life Cycle Database (ELCD), Chinese Life Cycle Database (CLCD), GaBi Databases, NREL-USLCI Database, etc. The resource consumption and environmental emission of unit mass materials in the raw material acquisition stage, unit power consumption in the production and manufacturing stage, unit oil consumption in the transportation stage, and materials with different disposal methods in the scrapping stage can be obtained from these databases.
- Material list database: mostly utilizing the name and unit of the International Reference Life Cycle Data System (ILCD), and adding the material list according to the characteristics of the wind turbine.
- Enterprise information database: storing the enterprise information involved in the life cycle of the wind turbine, including enterprise name, organization code, enterprise identification, identification rules, enterprise address, legal person information, responsible person information, enterprise website, and other fundamental data of the enterprise. The information of the database is obtained through enterprise registration and filing mainly.
- Product information database: storing the fundamental data, operation and maintenance data, and environmental emissions and energy consumption data in the life cycle of wind turbines. The fundamental data contain the structural information of all kinds of wind turbines and defines the structural relationship between the wind turbine and its components and between the components. In this database, the fundamental data are mainly obtained through integration with EIS, and the operation and maintenance data and environmental emissions and energy consumption data are obtained by real-time monitoring of IoT technology. Based on the database, the usability and real-time performance of LCA can be significantly improved.

#### 3.5.4. Inventory Data Processing Module

**Figure 6:**
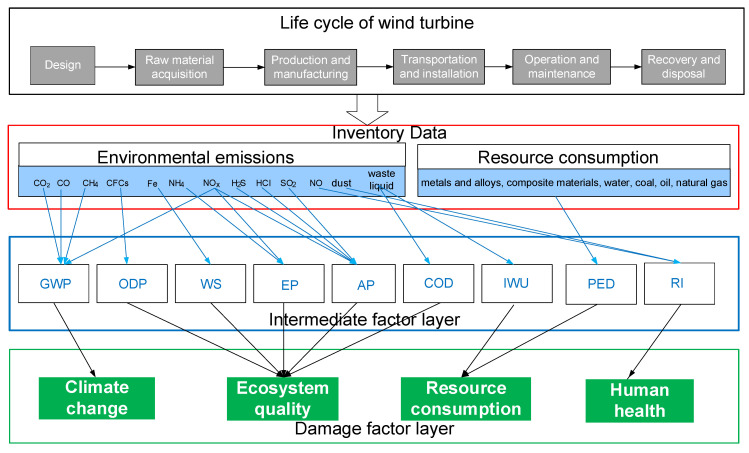
An LCA model for wind turbines based on IMPACT 2002+.

The Inventory Data Processing Module is the core module of the LCA platform. Based on the data support of material flow, energy flow, unit process, and database module, the module can quantify and analyze the data between input and output of resources and environment in each stage through online modeling and the corresponding algorithm. In this module, life cycle impact assessment (LCIA) index management and online modeling are included, which are as follows:

- LCIA index management: it mainly stores the types of evaluation indicators, conversion coefficients between different types of indicators, and environmental impact assessment standards of relevant industries or regions. It supports the calculation of LCIA characteristic indicators, normalized indicators, and weighted comprehensive indicators. Consequently, it is an important module for data normalization and environmental impact factor unification.
- Online modeling: Based on linking all types of life cycle inventory results via 14 midpoint categories to four damage categories, a feasible approach combining midpoint and damage is proposed, which is the IMPACT2002+ methodology [8]. Based on the IMPACT 2002+, the LCA model for wind turbines is established, as shown in Figure 6. Compared with other methods, the proposed model reduces the complexity and uncertainty of the evaluation process and guides the next optimization.

As shown in Figure 6, nine indicators including GWP, WS, EP, AP, COD, RI, IWU, ODP, and PED are selected for analysis in the intermediate factor layer. Additionally, climate change, ecosystem quality, resource consumption, and human health are the final index in the damage factor layer.

Since the previous data collection is aimed at each unit process, in order to ensure the accuracy and integrity of the evaluation results, it is necessary to determine a measurement reference of data before data processing, which is the function unit. In the raw material acquisition stage, the functional unit of the weight of various raw materials is kg. In the manufacturing stage, the functional unit of electrical energy consumption is kWh. In the transportation stage, the functional unit of the transportation distance and energy consumption are km and L separately. In the recovery and disposal stage, the functional unit of the weight of recycled or scrapped materials is kg.

The main influencing factors and functional units of each resource and environment index are shown in Table 4.

After the inventory analysis, in order to evaluate the accuracy of the inventory data, the relative error can be obtained by comparing the total mass of the wind turbine with the summing of the material quality of all components.

**Figure 7:**
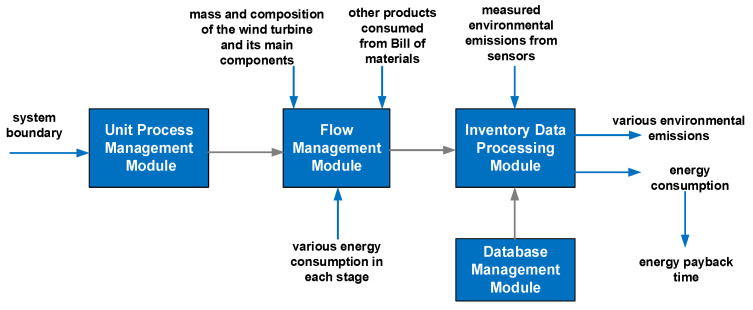
The data processing of the proposed platform.

To sum up, the data processing of the proposed platform is shown in Figure 7.

Based on the Unit Process Management Module, the decomposition of the wind turbine is completed. The quantity of various resource and energy consumption can be obtained from the Flow Management Module. Additionally, the total energy consumption and environmental emissions can be calculated, and all kinds of indicators can be characterized in the Inventory Data Processing Module, which is based on the parameters from the Database Management Module.

### 3.6. Application Layer

#### 3.6.1. Life Cycle Impact Assessment (LCIA)

LCIA is a process of quantitative or qualitative evaluation to obtain the performance of energy consumption and environmental emission after inventory analysis, and the inventory information will be translated into environmental impact scores. In terms of resource consumption of wind turbines, it mainly includes the evaluation of mineral energy consumption in the raw material acquisition stage, electric energy consumption in the production and manufacturing stage, and oil consumption in the transportation stage. For wind turbines, energy payback time (EPBT) is one of the key factors of evaluation, because it is one of the important indexes in the evaluation system of wind turbines generating capacity [43]. 

EPBT is the ratio of the total energy input of the wind turbine in the entire life cycle to the annual power generation during its operation [44], as shown in the following equation:

*EPBT* = *E*_*i*_/*E_0_* (5)

where *E_i_* is the total energy input of the wind turbine in its entire life cycle, and *E_0_* is the annual power generation of the wind turbine, which is calculated by

(6) $$E_{0}=365\times 24\times \bar{P}$$

where $\bar{P}$ is the average power of wind turbines under different wind speeds, and its calculation formula is

(7) $$\bar{P}=\int_{0}^{\infty }p(v)f(v)dv$$

where *p*(*v*) is the expression of wind turbines output power characteristic curve, and *f*(*v*) is the wind speed frequency distribution curve expression of wind turbines installation site.

In this study, the real-time wind speed and the actual output power of the wind turbine are collected through IoT technology. After obtaining the power characteristic curve of wind turbines and the wind speed frequency distribution of the installation site, *E_0_* will be calculated. At the same time, Ei is obtained according to the Inventory Data Processing Module. Thus, the EPBT of the wind turbine will be calculated. On this basis, according to the design life of the wind turbine, the power generation without emission in its life cycle can be calculated.

Besides, when the wind turbine parameters (starting wind speed, rated wind speed, maximum safe wind speed, rated power, maximum power, rated speed, working wind speed range, etc.) and installation address are known, the EPBT can be estimated in advance. On this basis, it can be predicted whether the installation site of wind turbines is appropriate, to avoid the loss caused by the improper installation scheme.

#### 3.6.2. Improvement Analysis

Improvement analysis is to analyze the results of the impact assessment, form a report, and provide practical improvement plans for different users. Enterprise users, government users, and third-party users are included in this system.

For enterprise users, according to the LCA results, the stage, or the key components with large resource consumption, energy consumption and environmental emission of wind turbines will be identified. Consequently, better raw materials can be selected. Additionally, design optimization, production process optimization, transportation route and vehicle optimization, operation and maintenance optimization, and recycling optimization can be carried out. For example, energy consumption in peak hours or idle time can be reduced.

For government users, through the results of impact assessment, the entire process of recycling can be monitored, the recovery rate of product components or materials can be improved, and the potential harm to the environment in the recycling can be reduced. Additionally, the suppliers of materials and components with the highest proportion of environmental emission can be list as the major concerns. Based on the evaluation results, relevant standards can be established, the corresponding evaluation mechanism can be improved, and the industrial structure will be optimized finally.

For the third-party users, the green certification will be accomplished by comparing the evaluation results with the defined green index value of the wind turbine.

## 4. Prototype System Implementation

Based on the proposed architecture, an LCA prototype system of the wind turbine has been developed. In this system, various users have different authority to manage data.

The main process of the life cycle assessment by using this system is illustrated as follows:

1. The enterprise registers in the system and inputs the information of name, identification, address, website, responsible person information, relevant qualifications, etc.
2. The decomposition of the wind turbine studied is determined, and the process flow is identified through the Unit Process Management Module. Additionally, then, the relevant parameters and the main information of key components for life cycle assessment will be input. Then, the material flow and energy flow information of each unit can be added, modified, deleted, and viewed through the Flow Management Module.
3. Based on the installation of various sensors, the relevant energy consumption and environmental emission data will be collected. Meanwhile, the operation data will be monitored, including wind speed, vibration, noise, engine speed, pitch angle, etc. According to these data, the output power characteristic curve is formed, which can be used to calculate the annual power generation of the wind turbine. Moreover, the real-time fault data and the fault rate curve of wind turbines are collected and formed. On this basis, the residual life prediction of the main components of wind turbines is carried out.
4. In addition to the real-time monitoring data, the Bill of Materials is acquired by integrating with the information systems of enterprises. Additionally, other data are obtained by the Database Management Module in the system.
5. Based on the online modeling and LCIA index management, the inventory data will be processed through the Inventory Data Processing Module. Based on inventory data, the energy consumption and environmental emission data at all stages of wind turbines are calculated systematically, and the evaluation results are analyzed visually. On this basis, the life cycle assessment report of wind turbines is formed.

## 5. Discussion

### 5.1. Workload and Accuracy of the Platform

#### 5.1.1. Comparison of the Workload

The workload of using LCA software include goal definition and scoping, data preprocessing, inventory analysis, and impact assessment. Compared with the existing software, the workload of data collection is greatly reduced, and most of the scoping of wind turbines has been established in detail in the proposed system.

To compare the LCA workload between the proposed system and the existing software, we suppose the total workload of data preprocessing, goal definition and scoping, inventory analysis, and impact assessment of the existing software are all 100%, and the weights of them on the LCA workload are 30, 20, 40, and 10%, respectively. The comparison results are shown in Table 5.

As shown in Table 5, the total workload of data preprocessing, goal definition and scoping, inventory analysis, and impact assessment of the proposed system are 48, 30, 80, and 100%, respectively. By weighting, the LCA workload of the proposed system is 62.4%, which means the proposed system effectively reduces the LCA workload of wind turbines.

#### 5.1.2. Estimation of the Accuracy

At present, the data collected by existing systems are often intertwined and need to be distributed among multiple parts, which is greatly affected by subjective factors. Therefore, the accuracy of the inventory data is not high. The proposed system can increase the data accuracy through IoT technology and improve the integrity of the data processing model through the integration with EIS.

As previously mentioned, the system boundary of LCA for wind turbines mainly includes six stages. In order to estimate the accuracy of the results, we assume that the accuracy of inventory analysis in each stage is increased by 5% on average. Consequently, the accuracy of the results is improved by (1 + 5%)^6^ × 100% = 134%

### 5.2. Characteristics of the Platform

The characteristics of the IoT-based life cycle assessment platform of wind turbines are summarized as follows:

#### 5.2.1. LCA of Wind Turbines Oriented

The platform is designed for wind turbines. In the process of the platform design, the corresponding system modules are established according to the life cycle process of the wind turbine. At the same time, according to the characteristics of the wind turbine, the LCA model based on the IMPACT2002+ is established. Additionally, the EPBT of wind turbines can be calculated accurately.

#### 5.2.2. Flexible Expansibility

To solve the lacking of information in the process of life cycle assessment, it can effectively integrate with the existing enterprise information systems through the ONS and DS based on IoT. Furthermore, the platform provides a standard application program interface and supports all kinds of users to develop and integrate various business applications on the platform. Therefore, it has the feature of extensibility. 

#### 5.2.3. Real-Time Data Updating

Based on the Internet of Things technology, real-time and dynamic data in the life cycle of wind turbines can be collected in the platform. Therefore, the databases of the platform have the function of updating and expanding, which ensures the objectivity and accuracy of the assessment results. 

#### 5.2.4. Effective Utilization of Data

Based on the big data technology, the platform can analyze the real-time data collected by sensors and obtained from EIS and has functions of intelligent monitoring, analysis, and prediction. Finally, it can serve the design and manufacturing enterprises of wind turbines, wind farm construction enterprises, and operation and maintenance enterprises, which include modeling service, evaluation service, simulation service, analysis service, and optimization service, etc. In the meantime, an open platform with controllable permissions can be provided through the construction of application components, so that the third party can participate in the application development and create business value.

#### 5.2.5. Ease of Use

The platform supports centralized user management, unified authentication and authorization, and business collaborative processing. Additionally, it is designed on B/S architecture, which is more convenient to operate and easier to maintain.

To sum up, the comparison between the proposed platform and several existing LCA systems is shown in Table 6.

## 6. Conclusions

In this paper, based on IoT technology, an LCA platform architecture for wind turbines is proposed. Additionally, object name service and directory service are used to provide external services in the network layer. In the meantime, an LCA model of wind turbines is established based on IMPACT 2002+. Therefore, the platform is wind-turbines-oriented and has good scalability and credibility.

The application of this platform can achieve effective integration and sharing with the existing enterprise information system and accomplish the real-time, intelligent collection of the energy consumption and environmental emission data generated in some stages. Given that the inventory data can be updated, the objectivity and accuracy of the assessment results can be ensured. It is of great significance for the sustainable development of the wind power industry.

In our future work, it is very important to deploy the LCA platform in more application scenarios. Additionally, combining the platform with blockchain technology to ensure the authenticity and credibility of data is also a key research direction. Besides, further research and exploration are needed to make the analysis results comparable with the existing systems.

## Figures and Tables

**Table 1:** The research objectives and scope of LCA for the wind turbine.

Users	Goal Definition	Research Object	System Boundary
Enterprises	Product development and improvement, green design	Wind turbine/main components	Raw material acquisition stage, production and manufacturing stage, recovery and disposal stage
The third-party certification department	Green certification	Wind farm/wind turbine/main components	Raw material acquisition stage, production and manufacturing stage, installation stage, operation and maintenance stage, recovery and disposal stage
Government	Standard-setting and policymaking	Wind farm/wind turbine	Design stage, raw material acquisition stage, production and manufacturing stage, transportation and installation stage, operation and maintenance stage, recovery and disposal stage

**Table 2:** The elementary raw materials in the wind turbine.

Components		Steel	Iron	Copper	Aluminum	Silicon	Glass Fiber	Epoxy Resin	Concrete
Tower frames	Fundamental ring	√							
	Tower drum	√							
Rotor	Blade						√	√	
	Hub		√						
	Pitch variation support						√	√	
Engine room	Shell						√	√	
	Framework	√							
	Generator	√		√		√			
	Gearbox	√		√	√				
	Principal axis	√							
	Transformer	√		√		√			
Foundation		√							√

**Table 3:** The main LCA influencing factors of the wind turbine.

Stage	Influencing Factor 1	Influencing Factor 2
Design stage	Design tool design software design drawings	Design test workshop waste disposal design waste disposal
Raw material acquisition stage	Metal material manufacturing mining smelting processing	Chemical products manufacturing chemical raw materials pretreatment chemical processing post-processing
Production and manufacturing stage	Communal facilities workshop office system	Manufacturing equipment machine tool special equipment testing equipment
Transportation and installation stage	Transportation type of shipping transportation route	Installation foundation construction tower hoisting engine room installation
Operation and maintenance stage	Installation test excavation of building materials building ingredients manufacturing transport operation of construction equipment	Maintenance repair overhaul component replacement
Recovery and disposal stage	Recovery disassembly and decomposition raw material extraction components recycling	Waste disposal incineration landfill

**Table 4:** Main influencing factors and functional units of resources and environmental indicators.

	Resource and Environment Indicators	Abbreviation	Influencing Factors	Functional Units
1	Global warming potential	GWP	CO_2_, etc.	kg CO_2_ eq
2	Solid waste	WS	steel, iron, glass fiber, etc.	kg
3	Eutrophication potential	EP	NH_4_, NO_x_, etc.	kg PO_4_^3−^ eq
4	Acidification potential	AP	SO_2_, NO_2_, H_2_S, HCl, etc.	kg SO_2_ eq
5	Chemical oxygen demand	COD	waste liquid draining	kg
6	Respirable inorganic	RI	dust, NO, etc.	kg PM2.5eq
7	Industrial water consumption	IWU	source of water	kg
8	Primary energy demand	PED	coal, oil, natural gas, etc.	MJ
9	Ozone depletion potential	ODP	CFCs	kg CFC-11eq

**Table 5:** Comparison of the LCA workload.

	Data Preprocessing			Goal Definition and Scoping	Inventory Analysis	Impact Assessment	the LCA Workload
	Data Collection	Data Analysis	Data Calculation				
The existing software	70%	10%	20%	100%	100%	100%	100%
The proposed system	20%	8%	20%	30%	80%	100%	62.4%
weights	30%			20%	40%	10%	100%

**Table 6:** Comparison of several LCA systems.

LCA System	Architecture	Data Sources	Real-Time Data Acquisition	Integration with EIS	Accuracy of Results	Wind Turbines Oriented
Gabi/Simapro/eBalance	C/S	Manual collection	N	N	Usual	N
The proposed platform	B/S	Automatic collection	Y	Y	High	Y

## Data Availability

Data sharing not applicable.
